# Pituitary Tumors in Maxillofacial Radiology and Daily Practice: A Scoping Review

**DOI:** 10.3390/dj14060368

**Published:** 2026-06-15

**Authors:** Lars Stucki, Uwe Mauer, Daniela Kildal, Noémi Katinka Rózsa, Margrit-Ann Geibel

**Affiliations:** 1 Faculty of Medicine and Dentistry, Danube Private University, AT-3500 Krems, Austria; 2 Faculty of Medicine, University of Bern, CH-3010 Bern, Switzerland; 3 Department of Neurosurgery, German Army Hospital Ulm, DE-89081 Ulm, Germany; 4 Department of Diagnostic and Interventional Radiology, University Hospital Ulm, DE-89081 Ulm, Germany; 5 Department of Radiology, Upper Valais Hospital Center Visp, CH-3930 Visp, Switzerland; 6 Department of Paediatric Dentistry and Orthodontics, Semmelweis University, H-1085 Budapest, Hungary; 7 Department of Oral and Maxillofacial Surgery, Dento-Maxillofacial Radiology, University of Ulm, DE-89081 Ulm, Germany

**Keywords:** oral and maxillofacial radiology, sella turcica, pituitary neoplasms, cone-beam computed tomography, lateral cephalogram

## Abstract

**Background:** Lateral cephalometric radiographs and large-field cone-beam computed tomography (CBCT) routinely used in orthodontics and maxillofacial radiology can reveal incidental pituitary tumors in the sellar region. Given the regular use of these imaging modalities, a structured overview of how pituitary tumors present on dental radiographs and how often they occur is clinically relevant. **Methods:** A scoping review was conducted according to PRISMA-ScR. MEDLINE via PubMed, Livivo, and Google Scholar were searched up to 20 January 2026 using MeSH terms and keywords for pituitary tumors and dental radiology. Human studies in English or German reporting on radiological presentation, clinical manifestation and epidemiology of pituitary tumors in the context of dental imaging were included. Study selection was performed independently by two reviewers. **Results:** Of 150 records, 15 studies were included: 2 case–control studies, 5 observational studies, 6 case reports, 1 questionnaire-based study and 1 neurosurgical guideline. Pituitary tumors most frequently presented with enlargement, deformation, or double contour of the sella turcica; growth hormone-producing adenomas additionally showed cephalometric changes such as mandibular and frontal sinus enlargement. The evidence is largely descriptive and does not permit robust estimates of prevalence or diagnostic accuracy but consistently identifies radiological “red flags” and recurrent clinical constellations, especially in acromegaly or unexplained craniofacial changes. **Conclusions:** Pituitary tumors, among the most common brain tumors, may first be suspected on routine dental radiographs. Distinct radiographic abnormalities combined with suggestive clinical features should prompt timely endocrine and neuroradiological evaluation, underscoring the need for heightened awareness among dental professionals.

## 1. Introduction

Pituitary tumors are brain tumors of the sellar region. They are the most common histologically diagnosed brain tumor in adolescents and young adults (AYAs) with an age-adjusted incidence rate of 4.47 per 100,000. Their incidence increases with age and they remain among the most common cerebral tumors across all age groups [1,2]. As they usually develop slowly, often causing only mild symptoms, incidental initial diagnosis through radiographs is not unusual [3,4]. As dentists regularly take radiographs of the sellar region using large-field cone-beam computed tomography (CBCT) scans or lateral cephalometric (LC) radiographs, they are likely to encounter such incidental findings [3,4,5].

Brain tumors are rare; however, incidental findings of abnormalities outside the primary area of interest are quite common in dental radiographs [2,6,7,8]. For instance, Edwards et al. noted that an enlarged sella turcica (ST) was among the most frequent significant incidental findings in CBCT scans [8]. Thus, it is important to thoroughly assess the sellar region in everyday practice. Although pituitary tumors in dental radiographs hold significant clinical importance, the available literature is limited mainly to case reports and small observational series.

Despite this clinical relevance, the literature is fragmented and heterogeneous, making a formal quantitative synthesis difficult. A scoping review is therefore an appropriate approach to systematically map the existing evidence, clarify key concepts, and identify gaps in knowledge.

The objective of this scoping review is to systematically map and summarize (1) how pituitary tumors may appear in dental radiographs, (2) their most common associated symptoms, and (3) relevant epidemiological patterns, in order to support early recognition and appropriate referral in daily dental practice.

## 2. Materials and Methods

The scoping review was registered with the International Prospective Register of Systematic Reviews (PROSPERO identifier: CRD420261353217). No deviations from the registered protocol occurred.

### 2.1. Study Design

This study was conducted as a scoping review, following the Preferred Reporting Items for Systematic Reviews and Meta-Analyses extension for Scoping Reviews (PRISMA-ScR). Given the anticipated heterogeneity and predominantly descriptive nature of the available evidence, a scoping rather than a quantitative systematic review was chosen to map key concepts, types of evidence, and research gaps. The intention was not to calculate the pooled prevalence or diagnostic performance of dental radiographs for pituitary tumors but to describe the spectrum of reported radiological signs and associated clinical contexts (Table S1).

### 2.2. Information Sources and Search Strategy

A comprehensive literature search was performed in MEDLINE via PubMed, Livivo, and Google Scholar from database inception to 20 January 2026. The search strategy combined MeSH terms and free-text keywords related to pituitary tumors and dental or maxillofacial radiology. The following search strategy was developed for PubMed and accordingly adapted for the other databases (Table S2): ((pituitary neoplasms[MeSH Terms]) OR (pituitary disease[MeSH Terms]) OR (pituitary gland/pathology[MeSH Terms]) OR (Craniopharyngioma[MeSH Terms]) OR (pituitary macroadenoma) OR (pituitary microadenoma) OR (pituitary incidentaloma)) AND ((cephalometry[MeSH Terms]) OR (Cone-Beam Computed Tomography[MeSH Terms]) OR (radiography, dental[MeSH Terms]) OR (radiography, panoramic[MeSH Terms]) OR (radiography, dental digital[MeSH Terms]) OR (lateral cephalo*) OR (CBCT)).

Reference lists of all included articles and relevant reviews were screened systematically for additional eligible studies (backward citation tracking). Duplicate records across databases were removed prior to screening using a combination of automatic detection (reference manager) and manual verification.

### 2.3. Eligibility Criteria

This review included human studies in English or German that reported on pituitary tumors or other lesions of the sellar region in relation to dental imaging (lateral cephalometric radiography, panoramic radiography, or CBCT). Studies were eligible if they contained information on at least one of the following domains: (1) radiological presentation on dental images, (2) clinical manifestations relevant for dental practice, or (3) epidemiology of pituitary tumors.

Studies that were not human studies or were not available in full text, English or German were excluded. Furthermore, articles that did not deal with pituitary neoplasms or dental radiographs were excluded.

### 2.4. Study Selection

The search was conducted independently by two authors (L.S. and M.G.). Titles and abstracts were screened using the eligibility criteria. Full texts of potentially relevant articles were then retrieved and assessed for inclusion. Disagreements were resolved through discussion and, if needed, consultation with a third reviewer.

### 2.5. Data Charting and Synthesis

A standardized data-charting form was used to extract information from the included studies, including study design, population characteristics, imaging modality, radiological features of the ST or pituitary region, clinical findings, and key epidemiological data. Data were synthesized narratively and organized around three main domains: radiological signs, epidemiology, and clinical manifestations pertinent to dental practice. Due to the heterogeneity and predominantly descriptive design of the included studies, no meta-analysis or formal assessment of diagnostic accuracy was undertaken.

## 3. Results

### 3.1. Search Outcome

**Figure 1:**
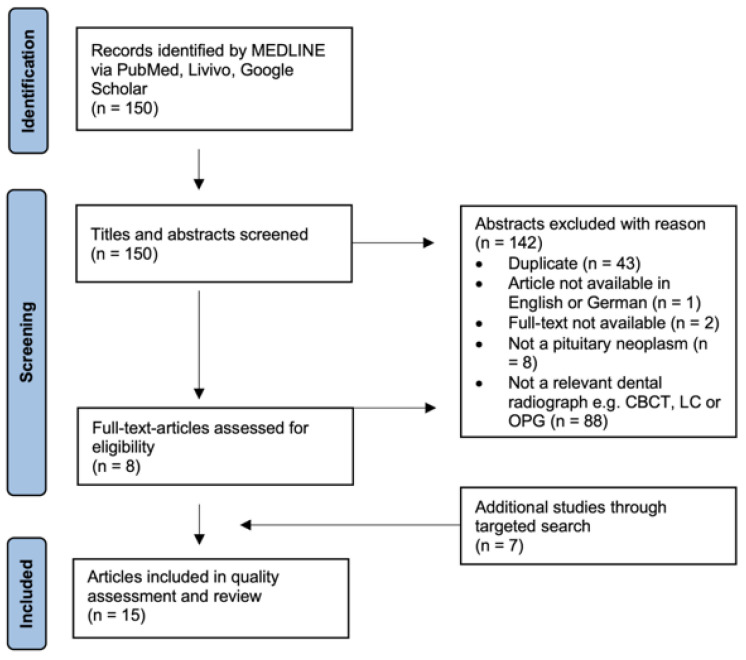
PRISMA-ScR Flow-chart.

Throughout the research period ending on 20 January 2026, a total of 150 potential articles were initially identified. Of these, 142 were excluded after applying the exclusion criteria. Additionally, 7 articles were added through a targeted search strategy. Consequently, the study incorporated a final selection of 15 articles (Figure 1).

### 3.2. Methodological Characteristics of Included Studies

Methodological limitations recurred across the included studies. First, the evidence base is dominated by single case reports, which, although often well documented, are inherently prone to selection and publication bias and cannot support estimates of incidence, predictive value, or causality. Second, even in the larger cross-sectional studies, patient selection was frequently retrospective and restricted to single centers, and follow-up beyond the initial radiological finding was rarely reported. Third, key potential confounders (e.g., age, sex, general health status) were only partially controlled for in some analytical studies, and clinical information such as endocrine status or visual field testing was inconsistently available.

As a consequence, the current literature predominantly only allows for a qualitative synthesis of typical radiological and associated clinical findings rather than robust quantitative statements. The available data do not permit firm conclusions about the probability that a given radiographic variation represents a clinically relevant pituitary tumor, nor about the natural history of incidentally detected abnormalities in dental radiographs. Nevertheless, the consistency of the reported patterns across multiple case reports and observational studies supports their use as “red flag” features that should prompt further clinical and radiological evaluation. The main characteristics and key findings of the included studies are summarized in Table 1.

### 3.3. Radiological Presentation on Dental Imaging

Pituitary tumors are typically characterized by expansive growth resulting in pressure on surrounding structures. This pressure can lead to a deviation of the adjacent bone, which manifests itself as radiological changes in the shape and general enlargement of the ST. The radiological presentation depends on the size and location of the tumor. ST changes can be interpreted in all planes when using CBCT scans, which provide comprehensive three-dimensional information about this anatomical structure. However, when using a lateral cephalogram, the ST can only be assessed in the sagittal plane, limiting the view to a two-dimensional perspective.

Regardless of the imaging plane, larger tumors can cause enlargement of the ST in all dimensions and directions [9,10,11,12,16]. Small tumors can cause bulges that extend into neighboring structures and may result in the downward displacement of the sellar floor into the sphenoid sinus [6,12,13,16]. If the tumor is located unilaterally, it may appear as a double contour in lateral radiographs [13]. Diligent differential diagnosis must be performed in this case, as the double sellar floor is also described as a normal shape variation or can be a result of incorrect positioning during radiography [19,20].

**Figure 2:**
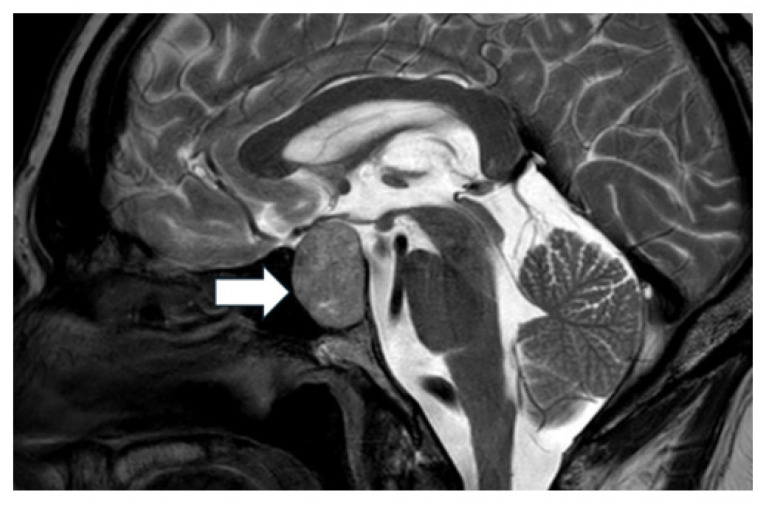
Sagittal T1-weighted contrast-enhanced MRI showing a pituitary macroadenoma (arrow) occupying and expanding the sella turcica with suprasellar extension.

**Figure 3:**
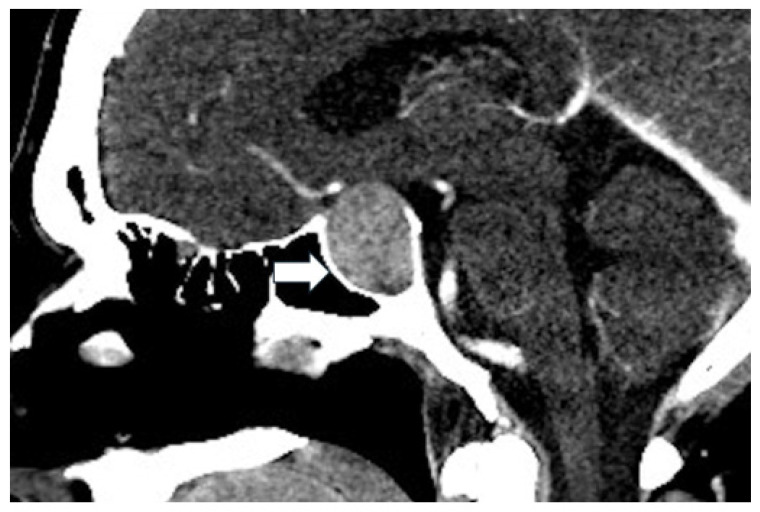
Sagittal CT image demonstrating enlargement of the ST (arrow) with inferior bulging of the ST floor in the sphenoid sinus, corresponding to the adenoma shown in Figure 2. The cortical bone remains intact.

**Figure 4:**
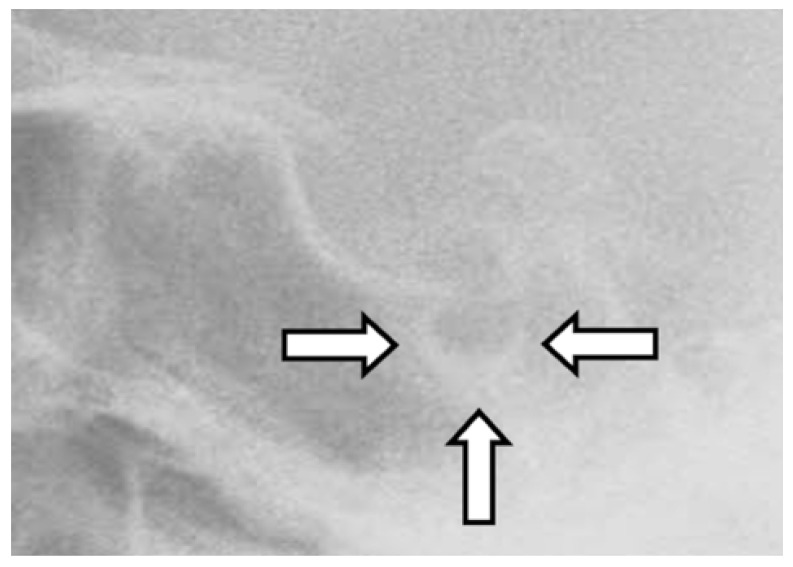
Displacement of the posterior sellar floor (arrow) in an LC image due to a pituitary adenoma.

As a consequence of the benign development of adenomas, the surrounding bone may appear thinner but usually remains intact in radiographs. Craniopharyngiomas, metastases, or other malignant entities, on the other hand, can lead to destruction of the surrounding bone due to their infiltrative character [3]. Figure 2 displays a pituitary adenoma on an MRI scan, while Figure 3 demonstrates the enlargement of the ST on the corresponding CT scan. An alteration of the anterior sellar floor due to a pituitary adenoma is shown in a lateral cephalogram in Figure 4. Notably, the bone appears intact in Figure 3 and Figure 4.

In patients with GH-producing pituitary adenomas, lateral cephalograms and CBCT demonstrate reproducible skeletal changes. Observational studies report enlargement of the ST (increased length and depth), increased mandibular length with predominant ramus elongation, increased lower facial height, and cephalometric patterns consistent with skeletal Class III patterns (reduced or negative ANB, increased SNB). Neurocranial alterations include enlargement of the frontal sinus and more prominent supraorbital ridges, as well as lengthening of the anterior and middle cranial fossae [9]. CBCT-based analyses additionally show increased mandibular volume, alterations in maxillary dimensions, and reduced upper airway cross-sectional areas at nasal, uvular, and mandibular levels [10]. These radiographic features have been observed consistently in acromegalic cohorts when compared with healthy controls and can support clinical suspicion of Growth-hormone (GH) excess in appropriate contexts.

### 3.4. Differential Diagnosis and Normal Variants of the Sella Turcica

Pituitary adenomas are by far the most common reason for tumorous changes in the ST [2,12]. However, various pituitary tumors, craniopharyngiomas, meningiomas, aneurysms of the internal carotid artery, Nelson’s syndrome, primary hypothyroidism, Rathke’s cleft cyst, empty sella syndrome, and a normal variation of the ST can also incite similar radiological changes [16,21]. All mentioned findings require further investigations and need to be diagnosed thoroughly.

Normal shape variations of the ST are important to consider, as there are nine different types in LC and CBCT scans [20]. A multinational meta-analysis of a total of 18,364 patients concluded that the normal ST shape is the most common one with a prevalence of 55.56%, followed by the ST bridge with 11.34%, irregularities of the posterior part of the dorsum sellae with 9.74%, oblique anterior wall with 9.55%, double contour of the floor with 6.89% and pyramidal shape of dorsum sellae with 6.6% [19]. During shape interpretation, it is important to consider that the ST grows with increasing age due to apposition in the tuberculum and resorption in the dorsal part and the floor of the ST [19,22].

Furthermore, the ST dimensions can be measured. According to Iskra et al., the average values are: ST length of 9.06 mm (standard error (SE) 0.15), depth of 8.0 mm (SE 0.13), and diameter of 11.15 mm (SE 0.17). It is important to note the large variance in ST dimensions among different studies [23]. For example, reported variations in ST depth range from 6.4 mm to 10.87 mm [19]. This variability limits the definition of universal cut-off values and underscores the need to interpret measurements in conjunction with clinical and other radiological findings. Despite the variability, reference values can offer guidance in differentiating abnormal development regarding ST size.

### 3.5. Epidemiology of Pituitary Tumors

Pituitary tumors are among the most common types of brain tumors, and are prevalent in almost all age groups. They account for a significant proportion of histologically diagnosed brain tumors: 17.7% in adults, 34.6% in adolescents (15–19 years), and 36.6% in adolescents and young adults (AYAs, ages 15–39) in the United States between 2016 and 2020 [2]. These tumors of the sellar region represent the most frequent histologically diagnosed brain tumors in AYAs, with an average annual age-adjusted incidence rate of 4.47 per 100,000 individuals [1].

Recent studies have shown that the prevalence of pituitary adenomas, the most common subtype of pituitary tumors, is higher than previously estimated. In Europe, cross-sectional studies have reported prevalence rates between 78 and 94 cases per 100,000 individuals [24,25]. In Argentina, the standardized incidence rate is 7.39 per 100,000 people per year [26]. Within pituitary adenomas, prolactinomas are the most common type found in Europe, Argentina, and the United States, followed by non-functioning adenomas and GH-producing adenomas [24,25,26].

The Central Brain Tumor Registry of the United States (CBTRUS) reports that females and Black individuals are more often affected by pituitary tumors [1,2]. The registry also highlights a 99.4% survival rate for these tumors, with children and young adults showing better survival outcomes, while survival rates tend to decrease with advancing age [4,5].

Although these epidemiological data are not specific to detection through dental radiographs, they underscore that pituitary tumors are relatively frequent among brain tumors and that incidental detection on imaging performed for dental or orthodontic purposes is plausible and clinically relevant.

### 3.6. Types of Pituitary Tumors and Clinical Manifestations

Tumors in the sellar region are predominantly benign neoplasms, primarily arising from the pituitary gland. These tumors are categorized into functioning and non-functioning types, each with distinct clinical manifestations.

A key concern with all pituitary tumors is their potential for growth, leading to compression of neighboring structures. Notably, 85% of macroadenomas (diameter > 10 mm) result in pituitary insufficiency, with symptoms arising depending on which hormonal axes are affected [16,27,28,29].

Expansive tumor growth can also impact cranial nerves involved in vision (II, III, IV, VI). Such compression can cause visual disturbances, including loss of visual fields or complete vision loss, due to optic nerve pressure. If the motor nerves (III, IV, VI) are compromised, patients may experience diplopia. Approximately 50% of macroadenomas lead to visual impairment, although this may be partially offset by compensation from the unaffected eye, often delaying clinical presentation [16,30,31].

The most prevalent pituitary tumor is the prolactinoma, which is characterized by elevated prolactin levels. Clinicians should note that increased prolactin can cause symptoms such as amenorrhea, galactorrhea, infertility, erectile dysfunction, and decreased libido [16,32].

Non-functioning adenomas are the second most common pituitary tumors after prolactinomas. Patients typically present with symptoms linked to the tumor’s mass effect, affecting adjacent structures. The severity of symptoms like pituitary insufficiency and impaired vision depends on tumor size and location [16,27,28,29,30,31].

GH-producing adenomas causing acromegaly are less common. Acromegaly is characterized by enlarged extremities, including hands, feet, nose, ears, lips, and the mandible, due to excessive GH [16,33]. In the craniofacial region, enlargement of the mandible, frontal sinus, and supraorbital ridges can be evident on LC and CBCT images.

Other functioning pituitary tumors are rare and exhibit a wide range of symptoms due to their histological diversity. For guidance on prevalence and symptoms, see the summarized evidence in Table 2 and Table 3.

## 4. Discussion

This scoping review mapped the current evidence on pituitary tumors in relation to dental radiographs, focusing on radiological signs, clinical manifestations, and epidemiological context relevant to orthodontists, oral and maxillofacial radiologists, and general dentists. As a scoping review, our intention was not to calculate the pooled prevalence or diagnostic performance of dental radiographs for pituitary tumors but to describe the spectrum of reported radiological signs and associated clinical scenarios.

Pituitary tumors are among the most common brain tumors in adolescents, young adults, and beyond [1,2]. Orthodontists and oral and maxillofacial surgeons have an increased chance of encountering manifestations of pituitary tumors because they frequently image the skull base with LC and large-field CBCT scans. Currently, there is a lack of comprehensive data regarding the prevalence of pituitary tumors diagnosed specifically through dental radiographs. However, research conducted by Edwards et al. has highlighted that an enlarged ST is one of the most common incidental findings observed in large-field CBCT scans [8].

In light of the limited and predominantly low-level evidence, our conclusions are intentionally conservative and focused on practical triage rather than definitive diagnostic rules. The reviewed case reports and observational studies suggest that many radiographic variations of the ST detected on dental images remain incidental, particularly when they occur in the absence of suggestive clinical features (e.g., visual disturbance, endocrine symptoms) and without clear mass-effect signs on advanced imaging. In contrast, combinations of radiographic abnormalities (marked enlargement or deformation of the sella, double sellar floor, bulging into adjacent structures) with clinical indicators such as new-onset mandibular prognathism, unexplained endocrinopathies, or visual complaints consistently characterized patients with clinically relevant pituitary tumors.

Investigations by Dostálová et al. and Tuncer et al. specifically examined the radiological characteristics of LC and CBCT scans in patients with GH-producing adenomas [9,10]. These studies consistently demonstrated significant enlargement of the ST, frontal sinus, mandible, and protrusion of the supraorbital ridge. Mandibular growth and the subsequent development of mandibular prognathism occurring after typical growth periods, such as in adulthood, should be considered a critical warning sign for the presence of these tumors. While studies have focused on GH-producing adenomas in LC and CBCT scans, other types of pituitary tumors remain less investigated despite their higher prevalence. This bias may be linked to the characteristic symptoms of acromegaly, such as skeletal class III malocclusion, which often necessitates orthodontic intervention. Nonetheless, since any type of pituitary tumor can grow slowly and can cause no, mild, or compensated symptoms, the first signs may be detected through dental radiography.

Incorporating an evaluation of general symptoms alongside radiological variations can enhance the suspicion of tumorous lesions. Nevertheless, given that clinical symptoms of pituitary tumors can be subtle and may only present in advanced stages of tumor progression, consultation with a specialist should be considered when encountering radiological alterations of the ST.

This review also emphasizes the limitations of the underlying evidence. Most available studies are case reports or small cross-sectional series, which are prone to selection and publication bias and do not allow for reliable estimation of prevalence, risk of progression, or diagnostic accuracy. Measurements of ST dimensions and shape variations show considerable variability across populations and imaging techniques, complicating the definition of universal cut-off values. Clinical follow-up is incompletely reported in many studies, so the long-term behavior of radiographically abnormal but clinically asymptomatic sellar findings remains uncertain. These constraints mean that incidental findings identified in dental radiographs should be interpreted as signals requiring clinical context and, when appropriate, further investigation, rather than as stand-alone diagnoses.

Given these limitations, practitioners should adopt a cautious yet vigilant approach when evaluating the sellar region in routine radiographs. Specific signs warranting further attention include enlargement or clear deformation of the ST, bulging into adjacent structures, and a double sellar floor that cannot be explained by positioning or known normal variants. When such radiographic findings occur in isolation and the patient is clinically asymptomatic, the current evidence suggests that many will remain incidental and may be managed with documentation and, if needed, non-urgent further imaging. However, if these findings are accompanied by clinical features such as mandibular prognathism developing outside the normal growth period, unexplained menstrual or sexual dysfunction, headaches, visual disturbances, or other endocrinological abnormalities, referral for specialized endocrine and neuroradiological assessment is warranted. Table 4 summarizes a pragmatic triage approach for ST findings on dental radiographs, integrating radiographic and clinical ‘red flags’ with suggested referral pathways.

Thus, although the certainty of evidence is low, this scoping review provides clinically usable guidance by helping clinicians distinguish situations in which an incidental radiographic change in the ST is likely to remain incidental from situations in which additional clinical evaluation and advanced imaging are indicated. Education and training in recognizing subtle morphological alterations are crucial for dental professionals to accurately differentiate physiological variants from pathological changes. Ultimately, heightened awareness and careful evaluation of radiological signs by dental professionals are essential steps toward early detection and improved outcomes for patients with pituitary tumors.

Future research should include prospective, multicenter studies evaluating incidental sellar findings on LC and CBCT scans, with standardized imaging protocols and systematic endocrine and ophthalmologic assessments. Such studies could clarify the true prevalence, natural history, and clinical significance of radiographic abnormalities of the ST detected in dental practice, and help define evidence-based thresholds for referral.

## 5. Limitations

The evidence base is dominated by case reports and small, often single-center observational studies with retrospective designs, incomplete follow-up, and limited control of confounders. No included study was designed to estimate the prevalence or diagnostic accuracy of dental radiographs for pituitary tumors, and reported frequencies of enlarged ST or suspected sellar pathology are sparse and inconsistent. Considerable inter-study variability in ST dimensions and shape classifications further limits the derivation of universal cut-off values. In keeping with the scoping methodology, the authors did not perform a formal risk-of-bias assessment or meta-analysis. Consequently, the findings of this review should be interpreted as a qualitative synthesis aimed at raising awareness and guiding triage, rather than as a basis for definitive diagnostic thresholds or practice guidelines.

## 6. Conclusions

Within the limitations of the predominantly descriptive literature identified in this scoping review, pituitary tumors can cause notable alterations of the ST on dental radiographs, including enlargement, deformation, and, in some cases, a double sellar floor. GH-producing adenomas may additionally present with characteristic craniofacial changes, such as mandibular prognathism, that often bring patients to orthodontic attention. Given that pituitary tumors are among the most common brain tumors, dental professionals should remain vigilant for these radiological changes and for clinical “red flags” suggestive of endocrine or visual dysfunction.

While many isolated, asymptomatic ST variations are likely incidental, our synthesis supports referral for specialized endocrine and neuroradiological assessment when suspicious radiographic findings coincide with suggestive symptoms. Incidental sellar findings on dental radiographs should therefore be regarded as prompts for clinical correlation rather than definitive diagnoses. Future prospective, well-designed studies are needed to better define the prevalence, natural history, and diagnostic value of incidental sellar abnormalities in dental imaging.

## Figures and Tables

**Table 1:** Overview of included studies on pituitary tumors and sella turcica findings in dental radiographs.

Author	n	Study Type	Pituitary Tumor Type	Imaging Modality	Relevant Findings
Dostálová et al. 2003 [9]	224	Case–control study	GH-producing adenoma (acromegaly)	LC	Orofacial skeleton: Increased facial height Ascending mandibular ramus elongated Ramus enlarged more than mandibular body Increased basion–supramental distance Negative difference between maxillary and mandibular protrusion Increased inclination angle of maxilla and lower gonial angle Maxilla NOT enlarged Neurocranium: Enlarged sella turcica Enlarged frontal sinus Prominent supraorbital ridge Control: healthy adults (n = 186)
Tuncer et al. 2014 [10]	43	Case–control study	GH-producing adenoma (acromegaly)	LC	Anterior and middle cranial fossae lengthened (*p* < 0.05) Posterior cranial fossa reduced in length, height, depth Sella enlarged (length and depth) (*p* < 0.001) Positive correlation between sella size and disease duration Increased frontal sinus width (*p* < 0.01) Increased height of atlas (*p* < 0.01) and axis (*p* < 0.05) Increased corpus dimensions and mandibular length Airways reduced at nasal, uvula and mandibular levels Control: healthy (n = 22)
Alkofide 2001 [3]	1	Case report		LC	Enlarged sella without bone destruction; cortical bone preserved Uniform enlargement with deepening of sella floor Narrowing and posterior displacement of dorsum sellae Sella malformation Differential: empty sella syndrome Craniopharyngioma
Gosau et al. 2009 [11]	1	Case report	GH-producing adenoma (acromegaly)	LC	Mandibular prognathism Prominent supraorbital ridge Enlarged sella
Chang et al. 2005 [12]	1	Case report	GH-producing adenoma (acromegaly)	LC	Sella enlarged in all dimensions with floor depression → anteroposterior (length) = 13 mm → craniocaudal (depth) = 12.2 mm Severe skeletal Class III (ANB = −8.1°) Mild maxillary retrusion (SNA = 80°) Marked mandibular prognathism (SNB = 88°) Severe anterior crossbite (negative incisor overjet: −11.5 mm)
Yang und Sung 2017 [13]	1	Case report	Prolactinoma	LC	Enlarged sella turcica Irregular bony contour of sella Double sellar floor
Klenke et al. 2022 [6]	300	Observational study	N/A	LC	Incidental findings: 33% require further diagnostic workup Adults more affected than children except in skull base, orbit, soft tissue Most findings located at skull base and caused by sella changes such as sella bridging or enlarged sella
Moffitt et al. 2011 [4]	201	Questionnaire based	N/A	LC	49.3% of surveyed orthodontists reported detecting a significant pathology during their career: 28% of significant pathologies were pituitary adenomas presenting as sella enlargement
Dogruel et al. 2022 [14]	100	Observational study	Acromegaly (n = 50) non-functioning adenoma (n = 20)	CBCT	Groups: A = acromegaly (n = 50); B = non-functioning pituitary adenoma (n = 20); C = healthy (n = 30) Significant differences: Mandibular length: A > B > C Maxillary length: A and C > B ANB: A > B > C SNB: A > B > C SNA: A and B > C Mandibular volume: A > B and C Nasal volume: A > B and C Alveolar ridge height: A > B and C
Pette et al. 2012 [7]	318	Observational study	N/A	CBCT	Incidental findings in 318 CBCT scans (max FOV 13 cm): 0.31% showed enlarged sella (1 case) 0.63% showed pituitary pathology (2 cases)
Edwards et al. 2014 [8]	427	Observational study	N/A	CBCT	Enlarged sella is among the most common significant incidental findings (3 cases)
Taner et al. 2019 [15]	80	Observational study	N/A	CBCT	Average values of the ST in healthy individuals with normal occlusion for differentiating pituitary pathologies: Length: 10.0 mm ± 1.7 mm; Depth: 9.0 mm ± 1.5 mm; Diameter: 12.2 mm ± 2.0 mm; Volume: 1026.6 mm^3^ ± 290.2 mm^3^
AWMF 2020 [16]		Guideline	N/A	N/A	Microadenoma: can lead to unilateral depression of the sella floor Macradenoma: penetration into the clivus and/or sphenoid sinus is possible, often leading to enlargement of the sella
Maret et al. 2014 [17]	1	Case report	Adenoma NOS	CBCT	Sella enlarged in all dimensions Layered appearance in dorsum sellae Excavated aspect in anterior sella wall under jugum sphenoidale
Koong 2019 [18]	1	Case report	Macrodenoma	CBCT	Lobular mass at the outer border Infiltrates/extends into/through the basosphenoid/occipital bone via the sella floor

Abbreviations: LC: Lateral cephalogram; CBCT: Cone-beam computed tomography; N/A: Not applicable; NOS: Not otherwise specified.

**Table 2:** Most common pituitary tumors and their prevalences and symptoms (according to [16,33,34,35,36,37,38]).

Tumor	Prevalence	Symptoms
Prolactinoma	32–66%	Women: amenorrhea, galactorrhea, loss of libido, Infertility Men: erectile dysfunction, loss of libido, Infertility
Non-functioning adenoma	25–30%	Pituitary insufficiency, loss of visual fields, loss of vision, double vision
Growth-hormone-producing adenoma	8–16%	Acromegaly: enlarged acres (hands, feet, nose, lips, mandible), enlarged internal organs and unspecific symptoms like snoring, carpal tunnel syndrome, joint problems and heavy sweating
ACTH-producing adenoma	2–6%	Morbus Cushing: proximal accentuated myopathy, Plethora, vascular fragility, bleeding tendency, trunk accentuated obesity, thin skin and stretch marks, diabetes, high blood pressure and hirsutism in women
TSH-producing adenoma	-	Hyperthyroidism, tachycardia, weight loss and heavy sweating
FSH-/LH-producing adenoma	-	Usually asymptomatic, rarely amenorrhea or hypogonadism

**Table 3:** Symptoms of pituitary insufficiency (according to [16,33,34,35,36,37,38]).

Insufficient Pituitary Hormone Axis	Prevalence	Symptoms
Somatotropic axis	>80%	Reduction in performance and quality of life
Gonadotropic axis	>80%	Women before the menopause: menstrual cycle disorders and amenorrhea Men: loss of libido and erectile dysfunction
Corticotropic axis	20–50%	Weakness, fatigue, weight loss, pallor and anemia SIDAH: nausea, headache and vomiting. Pronounced hyponatremia can lead to impaired consciousness, myoclonia and epileptic seizures Addison crisis: abdominal pain, nausea, vomiting, hypovolemia, hypotension, hypoglycemia and hyponatremia
Thyrotropic axis	20–50%	Hypothyroidism: weight gain, fatigue, dry and shaggy hair, constipation, tendency to freeze and edema formation

**Table 4:** Suggested triage of ST findings on dental radiographs [14,15,17,18].

Dental Imaging and Clinical Assessment	Suggested Interpretation	Recommended Action and Referral
Marked ST enlargement and/or deformation (e.g., clear increase in size, bulging into sphenoid sinus, irregular excavation of floor or walls) ***and*** clinical “red flags” (e.g., new/progressive mandibular prognathism after growth completion, headaches, visual complaints, menstrual or sexual dysfunction, other endocrine symptoms)	High suspicion of clinically relevant pituitary or sellar pathology	Prompt referral to an endocrinologist and/or specialized pituitary/neuroendocrine center; request dedicated pituitary MRI and, where available, neuroradiology review
Radiographic abnormality of the ST (as above) but ***no*** obvious clinical symptoms	Possible incidental sellar lesion or normal variant; clinical relevance uncertain	Document finding in the report; recommend non-urgent evaluation by the patient’s primary care physician or an endocrinologist for clinical assessment and consideration of pituitary MRI
ST shape or size variation within reported normal ranges (e.g., known normal variants such as sella bridge, double contour, pyramidal dorsum) and no clinical “red flags”	Likely normal anatomical variant	Record as normal variant; no immediate further work-up required. Consider comparison with prior or future imaging if available

## Data Availability

No new data were created or analyzed in this study. Data sharing is not applicable to this article.

## Supplementary Materials

The following supporting information can be downloaded at: https://www.mdpi.com/article/10.3390/dj14060368/s1, Table S1: Preferred Reporting Items for Systematic reviews and Meta-Analyses extension for Scoping Reviews (PRISMA-ScR) Checklist; Table S2: Search queries.

## Abbreviations

The following abbreviations are used in this manuscript:

CBCT	Cone-beam computed tomography
LC	Lateral cephalogram
ST	Sella Turcica
GH	Growth hormone
TSH	Thyroid-stimulating hormone
ACTH	Adrenocorticotropic hormone
LH	Luteinizing hormone
FSH	Follicle-stimulating hormone

## References

[1] Ostrom Q.T., Price M., Neff C., Cioffi G., Waite K.A., Kruchko C., Barnholtz-Sloan J.S. (2022). CBTRUS Statistical Report: Primary Brain and Other Central Nervous System Tumors Diagnosed in the United States in 2015–2019. Neuro Oncol..

[2] Ostrom Q.T., Price M., Neff C., Cioffi G., Waite K.A., Kruchko C., Barnholtz-Sloan J.S. (2023). CBTRUS Statistical Report: Primary Brain and Other Central Nervous System Tumors Diagnosed in the United States in 2016–2020. Neuro Oncol..

[3] Alkofide E. (2001). Pituitary Adenoma: A Cephalometric Finding. Am. J. Orthod. Dentofac. Orthop..

[4] Moffitt A.H. (2011). Discovery of Pathologies by Orthodontists on Lateral Cephalograms. Angle Orthod..

[5] Weisberg L.A., Zimmerman E.A., Frantz A.G. (1976). Diagnosis and Evaluation of Patients with an Enlarged Sella Turcica. Am. J. Med..

[6] Klenke D., Santander P., Vehring C., Quast A., Sommerlath Sohns J., Krohn S., Meyer-Marcotty P. (2023). Prevalence of Incidental Findings in Adult vs. Adolescent Patients in the Course of Orthodontic X-Ray Diagnostics. J. Orofac. Orthop..

[7] Pette G.A., Norkin F.J., Ganeles J., Hardigan P., Lask E., Zfaz S., Parker W. (2012). Incidental Findings from a Retrospective Study of 318 Cone Beam Computed Tomography Consultation Reports. Int. J. Oral Maxillofac. Implant..

[8] Edwards R., Alsufyani N., Heo G., Flores-Mir C. (2014). The Frequency and Nature of Incidental Findings in Large-Field Cone Beam Computed Tomography Scans of an Orthodontic Sample. Prog. Orthod..

[9] Dostálová S., Šonka K., Šmahel Z., Weiss V., Marek J. (2003). Cephalometric Assessment of Cranial Abnormalities in Patients with Acromegaly. J. Cranio-Maxillofac. Surg..

[10] Balos Tuncer B., Canigur Bavbek N., Ozkan C., Tuncer C., Eroglu Altinova A., Gungor K., Akturk M., Balos Toruner F. (2015). Craniofacial and Pharyngeal Airway Morphology in Patients with Acromegaly. Acta Odontol. Scand..

[11] Gosau M., Vogel C., Moralis A., Proff P., Kleinheinz J., Driemel O. (2009). Mandibular Prognathism Caused by Acromegaly—A Surgical Orthodontic Case. Head Face Med..

[12] Chang H.P., Tseng Y.C., Chou T.M. (2005). An Enlarged Sella Turcica on Cephalometric Radiograph. Dentomaxillofacial Radiol..

[13] Yang Y.-C., Sung C.-C. (2017). Double Sellar Floor Sign: A Clue of Pituitary Tumor. Intern. Emerg. Med..

[14] Dogruel F., Canger E.M., Gonen Z.B., Asantogrol F., Şahin A., Bayram F. (2022). The Evaluation of Changes in Maxillofacial Bones Using Cone Beam Tomography in Acromegaly. Med. Oral.

[15] Taner L., Deniz Uzuner F., Demirel O., Güngor K. (2019). Volumetric and Three-Dimensional Examination of Sella Turcica by Cone-Beam Computed Tomography: Reference Data for Guidance to Pathologic Pituitary Morphology. Folia Morphol..

[16] Deutsche Gesellschaft für Endokrinologie (2019). S2k-Leitlinie “Diagnostik und Therapie Klinisch Hormoninaktiver Hypophysentumoren”.

[17] Maret D., Telmon N., Treil J., Caron P., Nabet C. (2014). Pituitary Adenoma as an Incidental Finding in Dental Radiology: A Case Report. Ann. Intern. Med..

[18] Koong B. (2019). Atlas of Oral and Maxillofacial Radiology.

[19] Iskra T., Stachera B., Możdżeń K., Murawska A., Ostrowski P., Bonczar M., Gregorczyk-Maga I., Walocha J., Koziej M., Wysiadecki G. (2023). Morphology of the Sella Turcica: A Meta-Analysis Based on the Results of 18,364 Patients. Brain Sci..

[20] Jankowski T., Jedliński M., Grocholewicz K., Janiszewska-Olszowska J. (2021). Sella Turcica Morphology on Cephalometric Radiographs and Dental Abnormalities—Is There Any Association?—Systematic Review. Int. J. Environ. Res. Public Health.

[21] Jansen O., Forsting M., Sartor K. (2008). Neuroradiologie.

[22] Afrand M., Ling C.P., Khosrotehrani S., Flores-Mir C., Lagravère-Vich M.O. (2014). Anterior Cranial-Base Time-Related Changes: A Systematic Review. Am. J. Orthod. Dentofac. Orthop..

[23] Schwab J., Stucki L., Fitzek S., Tithphit A., Hönigl A., Stackmann S., Horn I., Thenner H., Dasser P., Woitek R. (2025). Radiological Assessment of Sella Turcica Morphology Correlates with Skeletal Classes in an Austrian Population: An Observational Study. Oral Radiol..

[24] Karavitaki N. (2012). Prevalence and Incidence of Pituitary Adenomas. Ann. Endocrinol..

[25] Daly A.F., Burlacu M.C., Livadariu E., Beckers A. (2007). The Epidemiology and Management of Pituitary Incidentalomas. Horm. Res..

[26] Day P.F., Loto M.G., Glerean M., Picasso M.F.R., Lovazzano S., Giunta D.H. (2016). Incidence and Prevalence of Clinically Relevant Pituitary Adenomas: Retrospective Cohort Study in a Health Management Organization in Buenos Aires, Argentina. Arch. Endocrinol. Metab..

[27] Fleseriu M., Bodach M.E., Tumialan L.M., Bonert V., Oyesiku N.M., Patil C.G., Litvack Z., Aghi M.K., Zada G. (2016). Congress of Neurological Surgeons Systematic Review and Evidence-Based Guideline for Pretreatment Endocrine Evaluation of Patients with Nonfunctioning Pituitary Adenomas. Neurosurgery.

[28] Nomikos P., Ladar C., Fahlbusch R., Buchfelder M. (2004). Impact of Primary Surgery on Pituitary Function in Patients with Non-Functioning Pituitary Adenomas? A Study on 721 Patients. Acta Neurochir..

[29] Raverot G., Assié G., Cotton F., Cogne M., Boulin A., Dherbomez M., Bonneville J.F., Massart C. (2015). Biological and Radiological Exploration and Management of Non-Functioning Pituitary Adenoma. Ann. d’Endocrinologie.

[30] Ntali G., Wass J.A. (2018). Epidemiology, Clinical Presentation and Diagnosis of Non-Functioning Pituitary Adenomas. Pituitary.

[31] Molitch M.E. (2014). Nonfunctioning Pituitary Tumors. Handbook of Clinical Neurology.

[32] Wong A., Eloy J.A., Couldwell W.T., Liu J.K. (2015). Update on Prolactinomas. Part 1: Clinical Manifestations and Diagnostic Challenges. J. Clin. Neurosci..

[33] Katznelson L., Laws E.R., Melmed S., Molitch M.E., Murad M.H., Utz A., Wass J.A.H. (2014). Acromegaly: An Endocrine Society Clinical Practice Guideline. J. Clin. Endocrinol. Metab..

[34] Mastantuoni C., Ugga L., Solari D., D’Aniello S., Spadarella G., Cuocolo R., Angileri F.F., Cavallo L.M. (2025). Prediction of Diabetes Insipidus Occurrence after Endoscopic Endonasal Removal of Sellar Lesions Using MRI-Based Radiomics and Machine Learning. J. Neurosurg. Sci..

[35] Naik C.S., Joshi S.S., Mhatre B.V., Garad A.R., Jain R.M. (2023). Use of High Condylectomy and Abdominal Dermis Fat Grafting for an Acromegaly Induced Bilateral Condylar Hyperplasia: A Case Report. Cureus.

[36] D’Onofrio G.F., Chiloiro S., Mattogno P., Lauretti L., Bianchi A., Olivi A., Cannavò S., Angileri F.F., Doglietto F. (2024). Endoscopic Transsphenoidal Surgery in Growth-Hormone Pituitary Adenomas (GH PitNETs): Current Indications, Limitations, and the Importance of a Multidisciplinary Approach. Front. Horm. Res..

[37] Zoli M., Angileri F.F., Cappelletti M., Doglietto F., Fiorindi A., Gianfreda C.D., Lauretti L., Massimi L., Musumeci A., Peron S. (2026). Endoscopic Endonasal Surgery for Prolactin-Secreting Adenoma: A Retrospective Multicenter Study by the Neuroendoscopy Section of the Italian Society of Neurosurgery. J. Neurosurg..

[38] Draghici R., Saracutu O.I., Pollis M., Manfredini D. (2026). Microprolactinoma (Pituitary Adenoma) as a Cause of Secondary Headache in a Pediatric Patient with Pain and Restricted Mouth Opening: A Case Report. Case Rep. Dent..

